# Supplementary material for: Assessment of Oxidative Stress-Induced Oral Epithelial Toxicity
Source: Biomolecules. 2023 Aug 11;13(8):1239. doi: 10.3390/biom13081239 (PMC10452318; doi:10.3390/biom13081239)

Supplementary Figure S1. Effect of H_2O_2 on cell morphology. Representative micrographs of oral keratinocytes treated with H_2O_2. H400, H357, and OKF6 cells were incubated with increasing concentrations of H_2O_2 (100-1200 μM) for 24 hours. At the end of the incubation period, morphological changes were assessed, and images were acquired using floid cell imaging system (FLoid™ Cell Imaging Station, Life Technologies Australia) with filter sets on the bright field at 20× magnification. Scale bar represents 100 μm and applies to all panels.

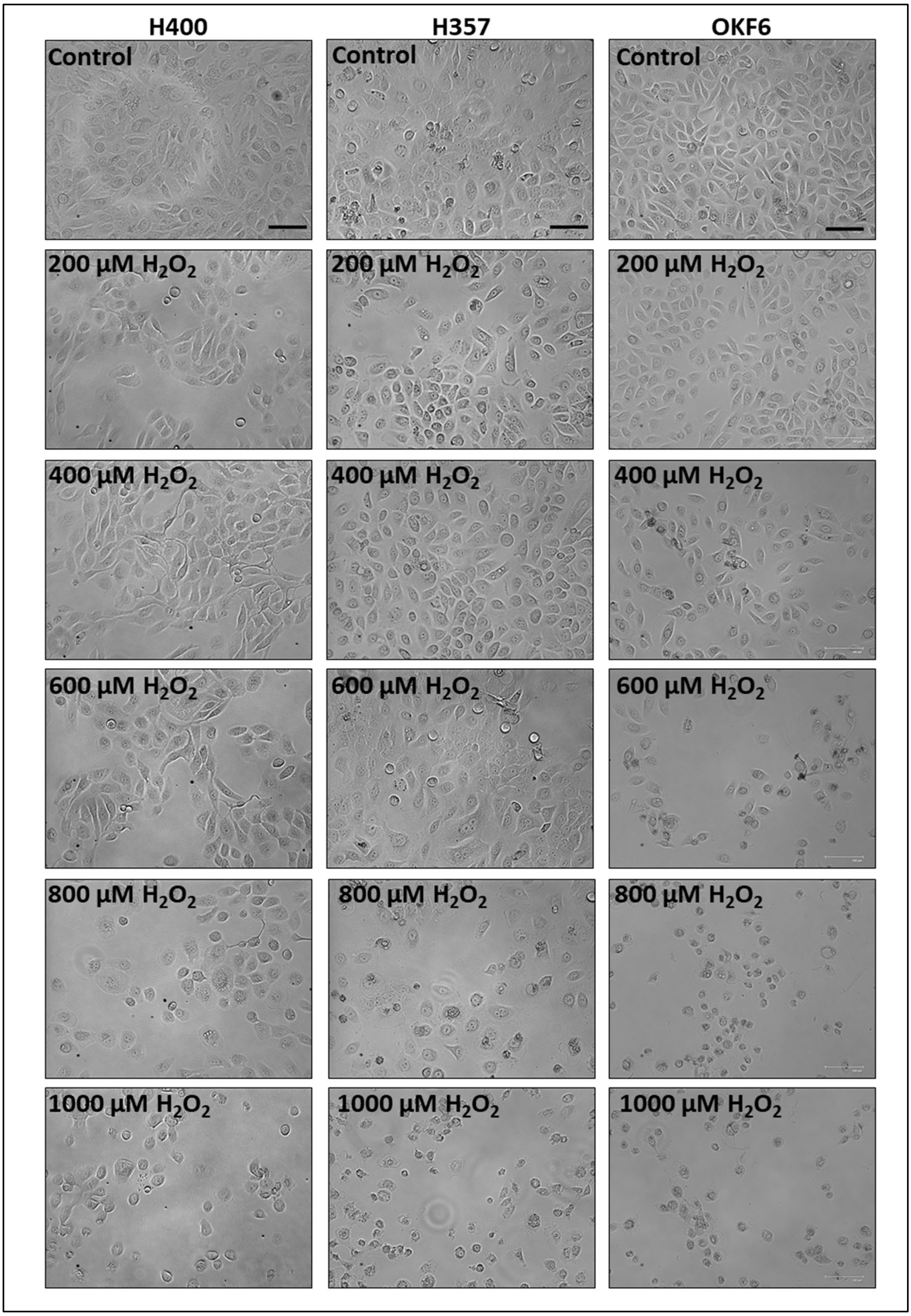

Supplementary Figure S2. H_2O_2 induced morphological alteration consistent with apoptotic features in oral epithelial cells. Bright field representative micrographs (20x) of OKF6 cells. **(a)**: Untreated (control) OKF6 cells retained their original morphology and were stably adherent to the culture plates. **(b)**: OKF6 cells after 24 hours treatment with an IC50 value of H_2O_2 (400 μM) displayed obvious apoptotic features, labelled with black arrows: A, apoptotic cells; C, condensed nucleus; F, fragmented nucleus; N, normal cell). Scale bar represents 100 μm and applies to all panels.

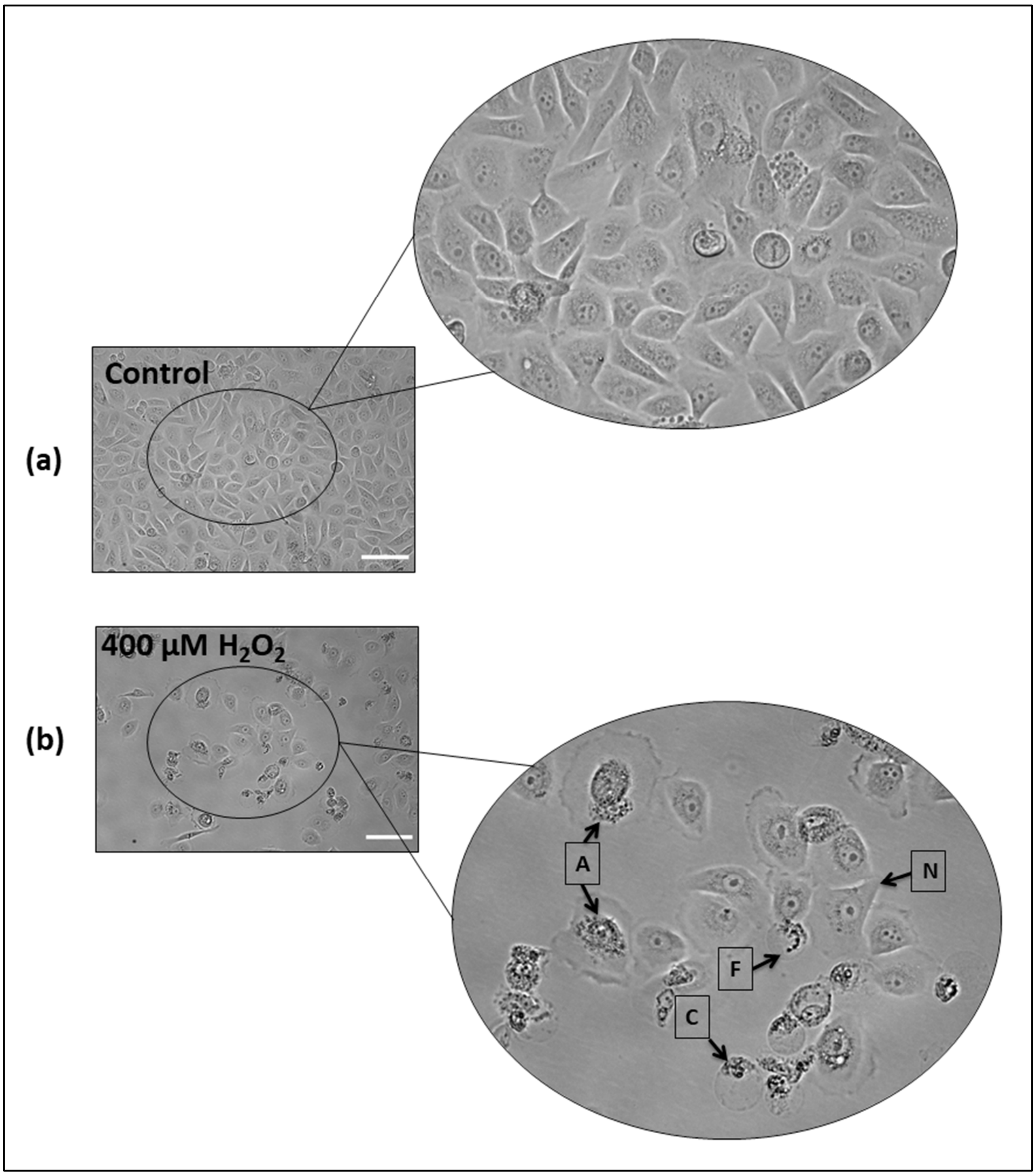

Supplement: Supplementary file 1 [file biomolecules-13-01239-s001.zip › biomolecules-2503139-supplementary.pdf]
